# Which Meso-Level Characteristics of Early Childhood Education and Care Centers Are Associated with Health, Health Behavior, and Well-Being of Young Children? Findings of a Scoping Review

**DOI:** 10.3390/ijerph18094973

**Published:** 2021-05-07

**Authors:** Raphael M. Herr, Katharina Diehl, Sven Schneider, Nina Osenbruegge, Nicole Memmer, Steffi Sachse, Stephanie Hoffmann, Benjamin Wachtler, Max Herke, Claudia R. Pischke, Anna Novelli, Jennifer Hilger-Kolb

**Affiliations:** 1 Mannheim Institute of Public Health, Social and Preventive Medicine, Medical Faculty Mannheim, Heidelberg University, 68167 Mannheim, Germany; Katharina.Diehl@medma.uni-heidelberg.de (K.D.); Sven.Schneider@medma.uni-heidelberg.de (S.S.); Nina.Osenbruegge@medma.uni-heidelberg.de (N.O.); nicole.memmer1@gmx.de (N.M.); jenny.hilgerkolb@gmail.com (J.H.-K.); 2 Institute of Psychology, University of Education Heidelberg, 69120 Heidelberg, Germany; sachse@ph-heidelberg.de; 3 Department of Public Health, Brandenburg University of Technology Cottbus-Senftenberg, 01968 Senftenberg, Germany; stephanie.hoffmann@b-tu.de; 4 Department of Epidemiology and Health Monitoring, Robert Koch Institute, 12101 Berlin, Germany; WachtlerB@rki.de; 5 Institute of Medical Sociology, Medical Faculty, Martin-Luther-University Halle-Wittenberg, 06112 Halle, Germany; max.herke@medizin.uni-halle.de; 6 Institute of Medical Sociology, Center for Health and Society, Medical Faculty, Heinrich-Heine-University Duesseldorf, 40225 Duesseldorf, Germany; Claudia.Pischke@hhu.de; 7 Chair of Health Economics, Technical University of Munich, 80992 Munich, Germany; anna.novelli@tum.de

**Keywords:** scoping review, early childhood education and care (ECEC) centers, kindergarten, young children, meso-level characteristics, health, health behavior, socioeconomic position, health inequalities

## Abstract

Characteristics of early childhood education and care (ECEC) centers might be relevant for children’s health. This scoping review aims to provide an overview of the association between meso-level characteristics (MLCs) of ECEC centers with children’s health, health behavior, and wellbeing. Five databases were searched for quantitative and qualitative research articles published in English or German since 1 January 2000 on health, health behavior, and wellbeing of children aged 0 to 6 years considering MLCs of ECEC centers. Two authors screened 10,396 potentially eligible manuscripts and identified 117 papers, including 3077 examinations of the association between MLCs and children’s health indicators (Kappas > 0.91). Five categories of MLCs were identified: (1) structural characteristics, (2) equipment/furnishings, (3) location, (4) facilities/environment, (5) culture/activities/policies/practices, and 6) staff. Only very few studies found an association of MLCs with body weight/obesity, and general health and wellbeing. Especially physical activity and mental health were related to MLCs. In general, the location (rural vs. urban, neighborhood status) seemed to be a relevant health aspect. MLCs of ECEC centers appeared relevant for child health indicators to different degrees. Future research should focus on these associations, in detail, to identify concrete ECEC indicators that can support health promotion in early childhood.

## 1. Introduction

An increasing number of children in economically developed countries are attending early childhood education and care (ECEC) facilities, such as childcare and daycare centers, family childcare homes, kindergartens, nurseries, and preschools [1]. In addition to the family as the primary agent of socialization, ECEC centers are the most important agent of socialization for the group of children in the preschool age [2] because children spend a considerable amount of time every day at these facilities. In ECEC centers, children make friends, eat together up to four meals per day, are physically active indoors and outdoors, and are in contact with peers from different social backgrounds (e.g., socioeconomic position of the family). Responsibilities of ECEC also include to maintain and promote the health of children in their care [3]. Attendance to ECEC has been found to be very beneficial for health, wellbeing and mental development for children [4,5]. However, it is not compulsory in some countries.

The theoretical framework for investigating the role of ECEC centers in the health of children aged 0–6 is provided by the social-ecological model established in the field of Public Health [6]. According to this framework, the concept of health is multifaceted and ranges from health-related behavior, such as physical activity and nutrition, to physical and mental health. ECEC centers are located at the meso-level, meaning between micro (the individual) and the macro (the society) level. The framework supports the assumption that characteristics of these meso-level characteristics (MLCs) are responsible for differences in the individual health, health behavior, and wellbeing of children [7]. Knowledge on the characteristics influencing health could help develop a health promoting environment, for instance, by architectural planning, the selection of equipment, interior design, and pedagogical training of personnel. For example, it is conceivable that the children will exercise to a higher amount if they have the opportunity, such as through a large outdoor area or animating equipment, or eat healthier if the employees are specially trained in nutrition.

MLCs include contextual and compositional aspects of ECEC centers. While contextual characteristics describe the structural conditions of an institution (e.g., equipment, location), compositional features (e.g., gender ratio, age ratio, proportion of children with immigrant background) merely represent aggregated information about the children attending the institution [8].

In order to be able to make general conclusions, it is necessary to summarize the individual characteristics into groups of MLCs. Contextual influences on physical activity, for example, can be grouped into physical, economic, political, and socio-cultural environments [9]. For ECEC centers, we assume that the relevant categories will be the following: structural characteristics, equipment/furnishings, the location of ECEC center (e.g., urban vs. rural region), facilities/environment, and culture and practices of the center.

To date, no comprehensive review that gives an overview on the association between children’s health and MLCs of ECEC centers exists [10]. Therefore, the aim of this review was to summarize which MLCs of ECEC centers are associated with health, health behavior, and wellbeing in children aged 0-6. In addition, we aimed to identify studies that further elucidate whether MLCs mediate or moderate the association between family’s socioeconomic position (SEP) and health in this age group. In view of the complexity of both, the characteristics at the meso-level and the health outcomes at the micro level, we decided to perform a scoping review to capture the current and comprehensive state of quantitative and qualitative research [11].

## 2. Materials and Methods

This scoping review follows the PRISMA-ScR (Preferred Reporting Items for Systematic reviews and Meta-Analyses extension for Scoping Reviews) Statement [12]. Ethical approval was not necessary because we only reviewed published manuscripts. The review was registered at Prospero (CRD42020161099) and the protocol was published recently [10].

### 2.1. Eligibility Criteria

To address the objectives of this scoping review, studies were included if they focused on health behavior, health, and wellbeing of children aged 0 to 6 years and took MLCs of ECEC centers into account. All manuscripts published in English or German since 1 January 2000 were considered for inclusion. Following the characteristics of scoping reviews, we included quantitative (cross-sectional, cohort, prospective, and case–control studies, as well as baseline data from intervention studies) and qualitative studies. We only included articles of studies that were conducted in economically developed countries (according to the United Nations classification) [13]. A detailed description of the inclusion and exclusion criteria and their respective rationales is presented in Table A1 and has been published previously [10].

### 2.2. Information Sources

We used PubMed/Medline, PsycINFO, Sociological Abstracts, Educational Resources Information Center (ERIC), and The Cochrane Library as databases for our literature search. The search took place on 2 December 2019.

### 2.3. Search Strategy

As described in our review protocol, the search strategy was first developed for PubMed/Medline and then adapted to the other databases [10]. Search terms were based on the Medical Subject Heading (MeSH) Thesaurus and complemented with additional relevant free-text terms. For example, we included search terms such as: pre-school*[Title/Abstract], kindergarten*[Title/Abstract], context[Title/Abstract], meso-level[Title/Abstract], caregivers[MeSH]), Pre-School Teacher[Title/Abstract], child-teacher relationship[Title/Abstract], classroom size[Title/Abstract], quality of care[Title/Abstract], playground[Title/Abstract], health[MeSH], quality of life[MeSH], dietary intake[Title/Abstract], meal times[Title/Abstract], physical activity[Title/Abstract], wellbeing[Title/Abstract]. The full search strategy can be found elsewhere [10].

### 2.4. Selection of Sources of Evidence

**Figure 1:**
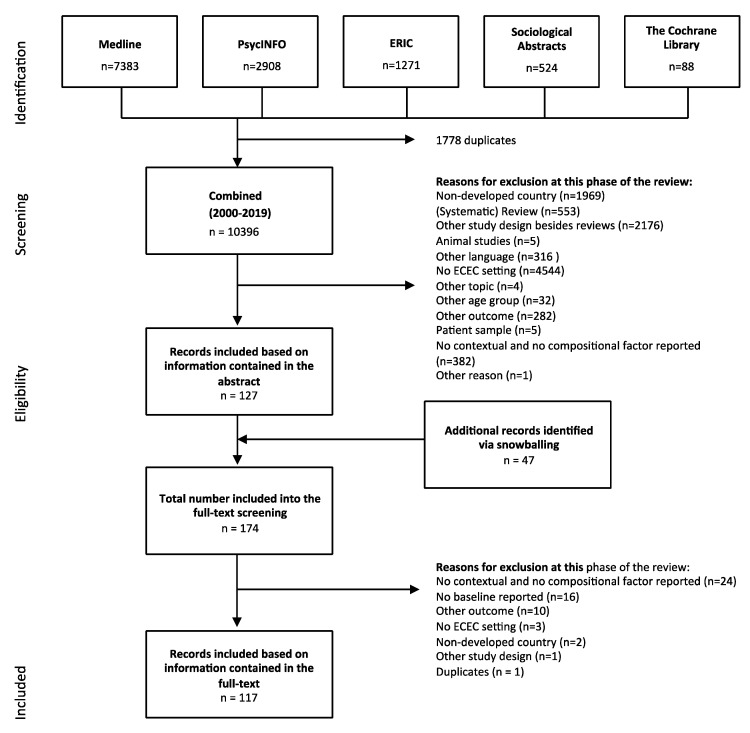
PRISMA flow diagram (according to the recommendations of Moher et al. 2009 for reporting reviews [15]).

After discarding duplicates, 10,396 potentially eligible manuscripts were found (Figure 1). First, title and abstract were screened, which yielded 127 manuscripts potentially eligible for inclusion in the review. The references of these manuscripts were screened as well (“snowballing”), yielding an additional 47 potentially eligible manuscripts. Second, these 174 manuscripts were reviewed in detail (full-text screening). Both selection steps were conducted independently by two reviewers (JH-K and KD), resulting in an excellent inter-rater agreement (first step: agreement = 99.9%, Cohen’s Kappa = 0.95; second step: agreement = 96.6%, Cohen’s Kappa = 0.91) [14]. In total, 117 manuscripts were included into this scoping review. Based on the 117 included studies, a total of 3077 examinations of the associations between MLCs of ECEC centers with children’s health, health behavior, and wellbeing were extracted and considered in our analysis.

### 2.5. Data Charting

Each study included in this scoping review was charted, using a standardized data extraction form, that had been tested by the team in previous studies [16,17]. Five of the authors charted data independently (RH, JH-K, NO, NM, KD). In addition, we conducted a double extraction of 5% of all included articles to ensure high data quality.

### 2.6. Data Items and Synthesis of Results

Data analysis and summary were conducted in three steps. First, a descriptive summary in the form of a table was created, including the following main data items of each included manuscript: author and year of publication, country of origin, study type and size, sample age, outcome main category, number of extracted examinations, and whether family SEP was reported (Table 1). In addition, we created figures to give an overview over the number of manuscripts dealing with the respective dependent variables (i.e., health, health behavior, and wellbeing) and the respective independent variables (i.e., contextual and compositional variables at ECEC center level). To be able to do this, we classified the independent variables into the following categories:

- Structural characteristics of ECEC center: e.g., amount of time in the institution, size of institution/groups/classroom, children to staff ratio, group composition/structure.
- Equipment/furnishings of ECEC center: e.g., fixed or mobile physical activity (outdoor, indoor) equipment/play environment, playground features (e.g., presence of sand pits, paddling pools, jumping equipment, slides, etc.).
- Location of ECEC center: e.g., urban vs. rural region, neighborhood SEP of institution.
- Facilities/environment of ECEC center: e.g., space (playground), noise, shadow, ventilation, years in operation.
- Culture/activities/policies/practices of ECEC center: e.g., time outside, health promotion activities, weather clothing policies, TV time, hygiene.
- Staff in ECEC center: e.g., competencies, educational level, specific training, attitudes, role model behavior, personality, teaching style, teacher-child-interaction, age, years in institution/childcare, BMI, race/ethnicity.
- Others.

In addition, we grouped the dependent variables into the following categories: (1) physical activity/sedentary behavior, (2) nutrition behavior, (3) physical health/development, (4) mental health/development, (5) body weight/obesity, (6) general health/wellbeing, and others.

In a second step, we mapped the evidence identified for associations between the respective dependent and independent variable categories by creating a graphical illustration depicting the kind of association (yes vs. no). The third step focused on whether SEP was considered and whether SEP had an influence.

## 3. Results

**Figure 2:**
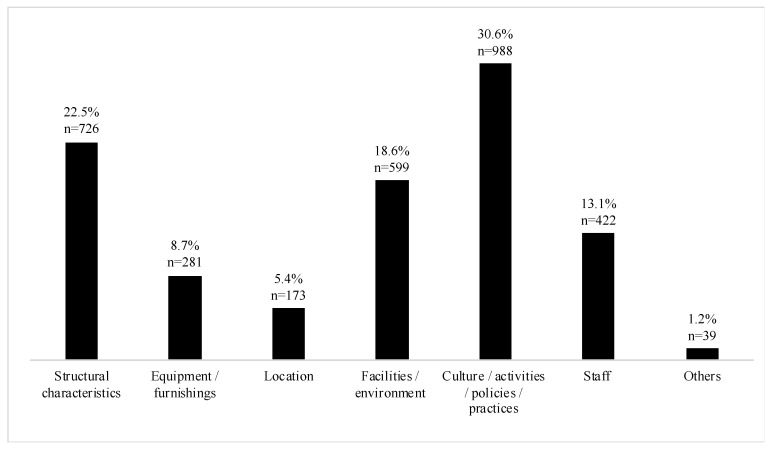
Frequency of ECEC center characteristics examined in the scoping review (%, *n*).

In the 117 included studies, 3077 examinations of the association between MLCs of ECEC centers with children’s health, health behavior, and wellbeing were identified. Culture of the ECEC center was the MLC most often examined (31%, *n* = 988, Figure 2), followed by structural characteristics of the ECEC center (23%, *n* = 726) and facilities/environment of the ECEC center (19%, *n* = 599). Potential associations with staff (13%, *n* = 422), equipment (9%, *n* = 281) and the location of the ECEC center (5%, *n* = 173) were least often studied.

**Figure 3:**
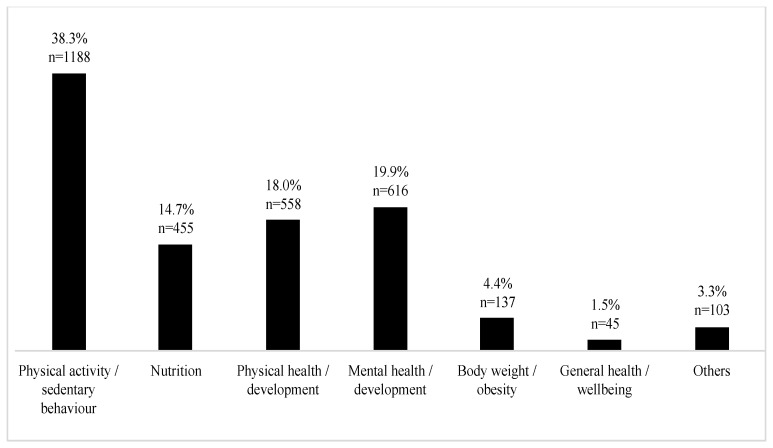
Frequency of health indicators examined in the scoping review (%, *n*).

Figure 3 specifies the frequency of the examined child health, health behavior, and wellbeing indicators of the included studies. The most often examined outcome was physical activity (38%, *n =* 1188), followed by mental health and development (20%, *n =* 616), physical health and development (18%, *n =* 558), and nutrition (15%, *n =* 455). Body weight/obesity (4%, *n =* 137), and general health/wellbeing (2%, *n =* 45) were studied least frequently.

**Figure 4:**
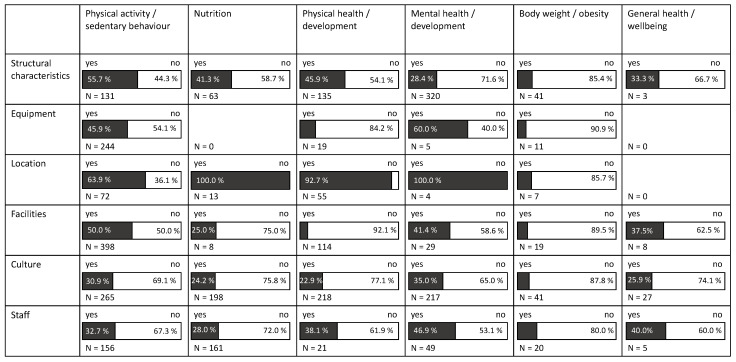
Associations between ECEC center MLCs with health, health behavior, and wellbeing of children.

Figure 4 presents the associations between early ECEC centers MLCs and the health, health behavior, and wellbeing of the children.

Physical activity of the children was most often examined in relation to the facilities of the ECEC (398 examinations), followed by the culture of the ECEC center (265 examinations), the equipment (244 examinations), the staff (156 examinations), and the structural characteristics of the ECEC center (131 examinations). Most often, an association was reported for the location (64% of 72 examinations), followed by the structural characteristics (56% of 131 examinations), the facilities (50% of 398 examinations), and the equipment (46% of 244 examinations). Associations with physical activity were found less often with the staff (33% of 156 examinations) and the culture (31% of 265 examinations) of the ECEC center.

Regarding the nutrition behavior of the children, the culture and the staff of the ECEC center was examined most frequently (198, and 161 examinations, respectively). The structural characteristics of the ECEC center was examined 63 times, the location 13, and the facilities 8 times. No study investigated the relation of the ECEC center equipment with nutrition behavior of the children. The most frequent association was found for the ECEC center structural characteristics (41% of 63 examinations). For staff, 28% of 161 examinations found an association, for facilities, it was 25%, and for culture 24%. For location, none of the 13 examinations reported an association with nutrition behavior.

The relationship between physical health and development and MLCs was studied 218 times for culture, 135 times for structural characteristics, 114 times for facilities, and far less often for location (55 times), staff (21 times), and equipment (19 times). The most frequent relation was found for location with 93% of the reported 55 associations. For ECEC center structural characteristics, 46% of 135 investigations reported a relation to physical health and development, followed by staff (38% of 21 examinations), culture (23% of 218 examinations), equipment (16% of 19 examinations), and only 8% of 114 examinations found an association with the facilities of the ECEC center.

The most frequently studied ECEC center determinant for mental health and development was structural characteristics of the institution (320 examinations). Culture was investigated 217 times, and staff, facilities, equipment, and location less frequently (49, 29, 5, and 4 times, respectively). Except for the ECEC center characteristics, that were examined very rarely (4-5 examinations) and reported a high frequency of associations (location 100%, equipment 60%), for staff, facilities, and culture the prevalence for an association was between 35 to 47%. For structural characteristics of ECEC centers, only 28% of the examinations found an association.

The association of body weight and obesity in children with the ECEC center characteristics were generally examined less frequently. Most often with culture and structural characteristics (41 examinations), followed by staff (20 examinations), facilities (19 examinations), equipment (11 examinations), and location (7 examinations). The number of associations was also limited, ranging from 20% for staff to 9% for equipment.

General health and wellbeing were also rarely examined in their association with ECEC center characteristics. No study investigated the association with equipment or the location. Three associations were examined with structural characteristics, five with staff, and eight with facilities. The relation with ECEC center culture reached 27 examinations, with only one quarter reporting an association.

The SEP was considered in 33 of the 117 included studies (28%). Of these, five studies (4% of total) reported an association or moderation between family SEP-indicators at the family level (e.g., household income, education of parents/mothers) with different MLCs at the ECEC center level, such as physical activity, naptimes, social behavior, impulsivity, or learning skills (Table A2). One study tested a potential moderation of SEP [29], and no study examined the role of MLCs on the association of SEP with health outcomes.

## 4. Discussion

This scoping review aimed to identify and synthesize findings on the association of MLCs of ECEC centers with health, health behavior, and wellbeing of children. 117 studies were included, yielding 3077 examinations. Regarding the diverse outcome indicators, a differentiated picture of the relevance of specific ECEC center characteristics for children’s health was found.

For physical activity/sedentary behavior, the location, the structural characteristics, the facilities, and the equipment of the ECEC centers appeared most relevant. However, for equipment, fewer than 50% of the examinations found an association (46%), albeit equipment, such as fixed or mobile physical activity equipment and playground features, could be regarded as a basic requirement for physical activity. The location was more relevant, indicating that whether an ECEC center was in an urban or rural neighborhood or the neighborhood SEP might be the most important meso-level factor for the physical activity of the children as identified by this review. A rural neighborhood, or a neighborhood with higher SEP, can be meaningful because there is a higher level of road safety, more outdoor space, and access to safe and bigger playgrounds [21,41,50]. However, and as also found by a previous literature review on physical activity and sedentary time in center-based childcare [133], a big variation in the measurement, reporting and degree of physical activity and sedentary time exits between studies, which might bias results.

For nutrition behavior, this review reveals that the location was most relevant, as all observations found an association of the location of the ECEC center with nutrition behavior. However, this association was investigated by only few examinations (13 times). The location of the ECEC center might be relevant for the children’s nutrition because children in socio-economically disadvantaged areas eat less vegetables and ECECs center in rural areas provide more vegetables [70]. Second most relevant was the structural characteristics of the ECEC center, but only 41% of the examinations found an association. The structural characteristics category comprises aspects such as childcare attendance, the children/staff ratio, the childcare type, or mixing ages within a childcare group. Even fewer investigations reported an association of nutrition behavior with ECEC center staff, facilities, or culture, despite these categories comprising aspects, such as staff eating the same lunch or the existence of a food program. Regarding potential interventions to increase healthy eating, a systematic review found that the consumption of fruits and vegetables could be influenced by healthy eating interventions, while effects on anthropometric change were inconclusive [134]. The study concluded that a single exposure strategy appears insufficient and that there needs to be an education component as well. By contrast, another review came to the conclusion that the influence of specific components of educators’ practices on children’s healthy eating remains inconclusive [135].

For physical health and development, the most frequent association was found for the location of the ECEC center (93% of the observations reported an association). Structural characteristics and staff were also more relevant, while few or very few associations with culture, equipment or facilities were reported. Especially the environment of the ECEC centers with aspects, such as size and quality of play area and the number and availability of play equipment, appeared less relevant for physical health and the development of the children.

For mental health and development of the children, staff, facilities, and culture appeared most important. However, only for equipment and location the number of observations found a relation in over 50% of the examinations and these categories were investigated very rarely (4 and 5 observations). For structural characteristics and culture most observations found no association with mental health/development of the children (65%, and 72%, respectively). Thus, according to our review, aspects, such as size or education type of ECEC center, as well as special programs or routines, seemed to have limited relevance for children’s mental health and development. However, another review found full-day kindergartens, compared with half-day kindergartens, to improve academic achievement and lifelong health, especially for children from lower SEP families [136]. By contrast, other reviews found very few associations between the child-staff ratios and staff education in preschool ECEC programs with children’s development [137,138]. In consequence, a heterogeneity not only among single studies, but also among review articles exits.

For body weight and obesity, the ECEC center characteristics appeared to have little relevance: for all categories, most observations found no association (between 85% and 91%). This indicates that the ECEC center’s role on children’s obesity might be limited. ECEC center measures, such as size of play area, the quality of the environment, time spent outdoor, staff participation in physical activity, and food programs seems to be helpful to a limited extent only. In general, childcare has not been reported to be protective for obesity [139].

For general health and wellbeing associations with ECEC center characteristics were also rather weak and seldomly examined. Further studies might investigate this relation in greater detail, applying particular instruments to assess the general health of young children.

Previous social epidemiology research found that child health is related to parental SEP [140]: Health behavior, prevalence of diseases, physical and mental health, wellbeing and other health outcomes were found to be poorer in children from socially disadvantaged families [141]. This raises the question, whether MLCs might mediate or moderate existing or emerging health inequalities. In this review, SEP was considered in only very few studies: Of these, only one study tested a potential moderation, while no study examined the role of MLCs with regard to the association of SEP with health outcomes. Further studies should try to close this research gap and map possible ways to alleviate health inequalities, as MLCs of ECEC centers might affect health, health behavior, and wellbeing above and beyond the individual-level. Changing these factors at the early childcare-level could be a strategy to reduce childhood health inequalities, as for young children (aged 0-6 years), ECEC centers are, next to families, the most important agents of socialization. From the perspective if life course epidemiology, early intervention via institutional factors could have a strong influence on future health inequalities over the life course.

### Strengths and Weaknesses of the Study

This study is the first systematic examination of the relevant research question whether MLCs of ECEC centers are associated with health, health behavior, and wellbeing of young children. However, some limitations have to be reported. A source of bias of this scoping review might be the varying weight of the different studies. From some studies, only one examination was extracted, while for others, many more examinations were extracted (i.e., up to 182 observations from the study of Loeb and colleagues [78]). It is, therefore, conceivable that some large studies might distort results in one direction and overemphasize some aspects, such as the location. Another limitation is the lack of comparability of the included studies, as the MLCs might differ by country and culture and different survey instruments were used to measure same aspects. In order not to widen the focus, any additional studies from economically developing countries and studies that were not published in German or English were excluded. However, it was our aim to include a wide range of studies from various countries and with different study designs, which is the nature of a scoping review. In addition, the structural characteristics of ECEC were highly variable. Among the studies included were both more traditional kindergartens with different lengths of care time and structured day care, as well as numerous other forms of institutionalized care. This might explain the different results, as the form of care might have different effects on health (e.g., on the diet of the children). Beside the broad scope of the review, another strength is that for quality reasons five percent of all studies were extracted twice and combined.

## 5. Conclusions

The results of this scoping review suggest that ECEC center characteristics are relevant for child health indicators to different degrees and reveals promising approaches for further research which appears vital to tackle health inequalities already in the first years of life. This review confirms the association of specific meso-level ECEC center characteristics with health, health behavior, and wellbeing. In addition, it provides information regarding which aspects at the meso-level account for this relationship. While only very few studies reported an association of MLCs with body weight/obesity, general health, and wellbeing, physical activity and mental health were related to MLCs. For physical activity the MLCs structural characteristics and location played an important role. Besides the location, the equipment was also associated with mental health/development of the children. In this context, the role of the SEP has mostly been insufficiently investigated in previous studies. When designing ECEC environments and planning prevention and intervention measures, this scoping review can help identify factors contributing to preschoolers’ health, health behavior, and wellbeing.

## Figures and Tables

**Table 1:** Overview over included studies.

Author (Year Published)	Country of Origin	Study Type/Design	Study Size (*n*)	Sample Age in Years (Mean; CV)	Outcome Main Category	Number of Extracted Examinations	SEP Reported (Yes, No)
Alexandrino et al., 2016 [18]	Portugal	Cross sectional study	152	2.6; 26.9	Physical health/development	30	no
Alkon et al., 2000 [19]	USA	Prospective study	360	3.7; x	Physical health/development	4	no
Andreyeva et al., 2018 [20]	USA	Cross sectional study	838	[3–5 years]; x	Nutrition behavior	42	no
Arhab et al., 2018 [21]	Switzerland	Cross sectional study	476	3.9; 17.9	Various outcomes	110	yes
Barandiaran et al., 2015 [22]	Spain	Cross sectional study	206	4.2; 12.8	Mental health/development	10	no
Barbosa-Cesnik et al., 2006 [23]	USA	Cross sectional study	198	1.8; x	Physical health/development	44	no
Bell et al., 2015 [24]	Australia	Randomized control study (baseline)	328	x; x	Physical activity/sedentary behavior	16	no
Belsky et al., 2007 [25]	USA	longitudinal study	1,364	x; x	Other	1	no
Blaine et al., 2015 [26]	USA	Cross sectional study	166	x; x	Nutrition behavior	95	no
Boldemann et al., 2006 [27]	Sweden	Cross sectional study	199	x; x	Various outcomes	27	yes
Bornstein et al., 2006 [28]	USA	Multimethod	113	x; x	Mental health/development	6	no
Bower et al., 2008 [3]	USA	Cross sectional study	x	[3–5 years]; x	Physical activity/sedentary behavior	18	no
Boyce et al., (2012) [29]	USA	Prospective study	338	5.3; 5.7	Other	6	yes
Brown et al., 2009 [30]	USA	Cross sectional study	372	4.2; 14.3–16.7	Physical health/development	6	no
Burchinal et al., 2010 [31]	USA	Cross sectional study	1,129	x; x	Mental health/development	8	yes
Byun et al., 2013 [32]	USA	Cross sectional study	331	x; x	Physical activity/sedentary behavior	5	yes
Campbell et al., 2000 [33]	Sweden	Cohort study	52	1.3; 18.2	Other	42	no
Cardon et al., 2008 [34]	Belgium	Cross sectional study	783	x; x	Physical activity/sedentary behavior	20	no
Carreiro-Martins et al., 2014 [35]	Portugal	Cross sectional study	3,186	3.1; 48.4	Physical health/development	29	no
Christian et al., 2019 [36]	Australia	Cross sectional study	678	3.4; 23.5	Various outcomes	30	yes
Coleman and Dyment 2013 [37]	Australia	Qualitative study	x	x; x	Physical activity/sedentary behavior	8	yes
Copeland et al., 2016 [38]	USA	Cross sectional study	388	4.3; 16.3	Physical activity/sedentary behavior	20	yes
Cosco et al., 2010 [7]	USA	Cross sectional study	53	x; x	Physical activity/sedentary behavior	78	no
De Decker et al., 2013 [39]	Belgium, Bulgaria, Germany, Greece, Poland, Spain	Qualitative study	87	x; x	Physical activity/sedentary behavior	12	no
De Schipper et al., 2003 [40]	The Netherlands	Cross sectional study	186	1.6; 37.0	Other	6	yes
De Craemer et al., 2014 [41]	Belgium	Randomized control study (baseline)	472	4.43; x	Physical activity/sedentary behavior	15	yes
Dettling et al., 2000 [42]	USA	Cross sectional study	61	3.7; x	Various outcomes	7	yes
Deynoot-Schaub and Riksen-Walraven 2006 [43]	The Netherlands	Longitudinal study	70	1.3; 3.0	Mental health/development	110	no
Dinkel et al., 2019 [44]	USA	Cross sectional study	49	x; x	Physical activity/sedentary behavior	15	no
Dörr et al., 2014 [45]	Germany	Randomized control study (baseline)	405	4.9; 16.3	Physical activity/sedentary behavior	30	yes
Dowda et al., 2004 [46]	USA	Cross sectional study	266	4.0; x	Physical activity/sedentary behavior	66	no
Dowda et al., 2009 [47]	USA	Cross sectional study	299	3.8; x	Physical activity/sedentary behavior	30	no
Dyment and Coleman 2012 [48]	Australia	Mixed-methods study	16	x; x	Physical activity/sedentary behavior	8	yes
Eichinger et al., 2017 [49]	Germany	Randomized control study (baseline)	735	4.8; 54.0	Physical activity/sedentary behavior	4	yes
Eichinger et al., 2018 [50]	Germany	Randomized control study (baseline)	735	4.8; 54.1	Physical activity/sedentary behavior	2	yes
Ek et al., 2019 [51]	Sweden	Qualitative study	15	x; x	Physical activity/sedentary behavior	11	no
Enserink et al., 2015 [52]	The Netherlands	Longitudinal study	ca. 1,600	x; x	Physical health/development	173	yes
Erinosho et al., 2016 [53]	USA	Cross sectional study	544	x; x	Physical activity/sedentary behavior	20	no
Fossdal et al., 2018 [54]	Norway	Cross sectional study	289	x; x	Physical activity/sedentary behavior	4	no
Frenkel et al., 2019 [55]	USA	Prospective study	75	4.0; x	Physical health/development	3	yes
Gagné and Harnois 2013 [56]	Canada	Cross sectional study	242	[3–5 years]; x	Physical activity/sedentary behavior	9	no
Goto et al., 2019 [57]	Japan	Cross sectional study	2,902	5.2; x	Body weight/obesity	7	no
Gronholt Olesen et al., 2015 [58]	Denmark	Cross sectional study	350	x; x	Physical activity/sedentary behavior	6	no
Gubbels et al., 2010 [59]	The Netherlands	Cohort study	2,396	x; x	Body weight/obesity	15	no
Gubbels et al., 2011 [60]	The Netherlands	Cross sectional study	175	2.6; x	Physical activity/sedentary behavior	10	no
Gubbels et al., 2012 [61]	The Netherlands	Cross sectional study	175	2.6; x	Physical activity/sedentary behavior	50	no
Gubbels et al., 2015 [62]	The Netherlands	Cross sectional study	398	2.3; 37.0	Nutrition behavior	44	no
Gubbels et al., 2018 [63]	The Netherlands	Cross sectional study	152	2.9; 26.3	Physical activity/sedentary behavior	24	no
Henderson et al., 2015 [64]	USA	Cross sectional study	389	4.7; x	Physical activity/sedentary behavior	35	yes
Hesketh and van Sluijs 2016 [65]	UK	Cross sectional study	201	x; x	Physical activity/sedentary behavior	72	yes
Himberg-Sundet et al., 2019 [66]	Norway	Randomized control study (baseline)	x	x; x	Nutrition behavior	87	yes
Hinkley et al., 2016 [67]	Australia	Cross sectional study	731	4.6; 15.2	Physical activity/sedentary behavior	9	yes
Hoffmann et al., 2014 [68]	Germany	Cross sectional study	434	4.9; 20.4	Body weight/obesity	2	yes
Hughes et al., 2007 [69]	USA	Cross sectional study	549	4.1; x	Nutrition behavior	20	no
Jones et al., 2017 [70]	Australia	Cross sectional study	49	x; x	Various outcomes	28	no
Kharofa et al., 2016 [71]	USA	Cross sectional study	349	4.3; 16.3	Nutrition behavior	21	yes
Koningstein et al., 2015 [72]	The Netherlands	Cohort study	852	x; x	Physical health/development	20	yes
Kotch et al., 2007 [73]	USA	Intervention study	388	x; x	Other	4	no
Lee et al., 2013 [74]	USA	Cohort study	4350	x; x	Various outcomes	35	yes
Lehto et al., 2019a [75]	Finland	Cross sectional study	586	4.7; 19.2	Nutrition behavior	21	yes
Lehto et al., 2019b [76]	Finland	Cross sectional study	586	4.7; 19.2	Nutrition behavior	23	yes
Linting et al., 2013 [77]	The Netherlands	Cross sectional study	103	2.4; 28.0	Other	6	no
Loeb et al., 2004 [78]	USA	Mixed-methods study	451	2.4; 32.6	Mental health/development	182	yes
Luchini et al., 2017 [79]	USA	Cross sectional study	50	[3–5 years]; x	Nutrition behavior	5	yes
Määttä et al., 2018 [80]	Finland	Cross sectional study	779	4.3; 19.2	Physical activity/sedentary behavior	17	yes
Määttä et al., 2019 [81]	Finland	Cross sectional study	778	4.3; 19.2	Physical activity/sedentary behavior	72	yes
Maggi et al., 2011 [82]	Vernon, Merritt, Kamloops	Cross sectional study	621	3.8; 18.4	Mental health/development	11	yes
Marr et al., 2003 [83]	single suburban–rural area of upstate New York	Cross sectional study	40	x; x	Other	5	no
Martensson et al., 2009 [84]	Sweden	Cross sectional study	198	5.3; 10.5	Other	4	yes
Mazzucca et al., 2018 [85]	USA	Cross sectional study	559	x; x	Physical activity/sedentary behavior	1	no
Mikkelsen 2011 [86]	Denmark	Cross sectional study	4200	x; x	Physical activity/sedentary behavior	2	no
Musher-Eizenman et al., 2010 [87]	USA	Cross sectional study	46	6.3; 36.5	Nutrition behavior	2	no
Nafstad et al., 2005 [88]	Norway	Cross sectional study	942	x; x	Other	99	no
NICHD 2000 [89]	USA	Mixed-methods study	1158	x; x	Mental health/development	180	yes
NICHD 2001 [90]	USA	Mixed-methods study	1140	x; x	Mental health/development	15	yes
Niemistö et al., 2019 [91]	Finland	Cross sectional study	945	5.4; 20.4	Other	70	yes
O’Connor and Temple 2005 [92]	Australia	Qualitative study	45	x; x	Physical activity/sedentary behavior	4	no
Olesen et al., 2013 [93]	Denmark	Cross sectional study	426	5.8; 5.2	Physical activity/sedentary behavior	10	no
Park et al., 2019 [94]	USA	Cross sectional study	129	3.6; 22.8	Body weight/obesity	7	no
Pate et al., 2008 [95]	USA	Cross sectional study	493	4.2; 16.7	Physical activity/sedentary behavior	4	no
Pate et al., 2014 [96]	USA	Cross sectional study	301	x; x	Physical activity/sedentary behavior	3	no
Peden et al., 2017 [97]	Australia	Cross sectional study	301	x; x	Physical activity/sedentary behavior	42	no
Ray et al., 2016 [98]	Finland	Qualitative study	x	x; x	Nutrition behavior	6	no
Roberts et al., 2016 [99]	USA	Cross sectional study	2203	4.0; 14.0	Mental health/development	8	no
Röttger et al., 2014 [100]	Germany, Switzerland, France	Cross sectional study	114	5.3; 12.3	Physical activity/sedentary behavior	1	yes
Roubinov et al., 2019 [101]	USA	Longitudinal study	338	5.3; 6.0	Mental health/development	2	yes
Schlechter et al., 2017 [102]	USA	Cross sectional study	73	x; x	Physical activity/sedentary behavior	2	no
Scott et al., 2018 [103]	USA	Cross sectional study	1551	4.5; 7.1	Other	12	yes
Siekkinen et al., 2013 [104]	Finland	Longitudinal study	1268	6.1; 4.6	Other	18	no
Slack-Smith et al., 2004 [105]	Australia	Prospective study	846	x; x	Other	3	no
Smith et al., 2016 [106]	USA	Cross sectional study	6125	x; x	Physical activity/sedentary behavior	20	no
Söderström et al., 2013 [107]	Sweden	Cross sectional study	172	x; x	Other	36	yes
Soini et al., 2014 [108]	Finland	Longitudinal study	81	x; x	Physical activity/sedentary behavior	4	no
Staiano et al., 2018 [109]	USA	Cross sectional study	104	3.3; 15.2	Physical activity/sedentary behavior	12	no
Stanton et al., 2003 [110]	Australia	Cross sectional study	49	x; x	Other	2	yes
Staton et al., 2015 [111]	Australia	Longitudinal study	168	4.9; 6.6	Other	3	yes
Stephens et al., 2014 [112]	USA	Cross sectional study	1352	3.4; x	Physical activity/sedentary behavior	19	yes
Stich et al., (2006) [113]	Germany	Cross sectional study	6420	6.0; 6.1	Other	12	no
Stich et al., (2017) [114]	Germany	Longitudinal study	14,068	5.9; 6.6	Various outcomes	24	yes
Sugiyama et al., 2012 [115]	Australia	Cross sectional study	89	4.1; 14.6	Physical activity/sedentary behavior	20	no
Sun and Sundell 2011 [116]	USA	Cross sectional study	2819	x; x	Physical health/development	39	no
Tandon et al., 2011 [117]	USA	Longitudinal study	8950	4.4; 0.2	Other	1	yes
Ross et al., 2013 [118]	USA	Intervention study	339	4.5; 6.7	Various outcomes	14	yes
True et al., (2017) [119]	USA	Cross sectional study	229	4.2; 16.7	Other	33	yes
Tucker and Irwin 2010 [120]	Canada	Intervention study	140	3.4; 23.4	Physical activity/sedentary behavior	1	yes
Tucker et al., (2015) [121]	Canada	Cross sectional study	218	4.2; 23.2	Physical activity/sedentary behavior	15	yes
Van Beeck et al., 2015 [122]	The Netherlands	Cross sectional study	2318	x; x	Other	3	no
Van Cauwenberghe et al., 2012 [123]	Belgium	Cross sectional study	573	5.4; 7.4	Physical activity/sedentary behavior	17	no
Van Stappen et al., 2018 [124]	Belgium, Bulgaria, Germany, Greece, Poland and Spain	Cross sectional study	3578	4.8; 8.3	Physical activity/sedentary behavior	1	yes
Vanderloo and Tucker 2017 [125]	Canada	Cross sectional study	113	4.7; 14.1	Physical activity/sedentary behavior	24	no
Vanderloo et al., 2014 [126]	Canada	Cross sectional study	31	4.107; 20.7	Physical activity/sedentary behavior	10	no
Vanderloo et al., 2015 [127]	Canada	Cross sectional study	218	4.2; 23.2	Physical activity/sedentary behavior	57	yes
Ward et al., 2017 [128]	Canada	Cross sectional study	723	4.0; 17.5	Nutrition behavior	53	no
Werner et al., 2015 [129]	The Netherlands	Cross sectional study	245	2.9; 22.6	Other	7	no
Wolfenden et al., 2011 [130]	Australia	Cross sectional study	764	3.9; 20.3	Body weight/obesity	1	yes
Zandvoort et al., 2010 [131]	Canada	Qualitative study	54	x; x	Physical activity/sedentary behavior	1	no
Zhang et al., 2018 [132]	Australia	Cross sectional study	274	1.6; 21.0	Physical activity/sedentary behavior	48	yes

x = not reported; [] = age range if mean was not reported; Age in month was converted to years and multiple data were calculated as mean; CV = coefficient of variation in % (standard deviation/mean x 100).

## Appendix A

**Table A1:** Inclusion and exclusion criteria.

	Included	Excluded
Study designs	► Cross-sectional studies	► Case studies
	► Cohort studies	► Cell studies
	► Prospective studies	► Reviews
	► Case–control studies	► Author replies/comments
	► Qualitative studies	► Animal studies
	► Intervention studies (only baseline data)	
Populations	► Children aged 0-6 years attending an early childcare facility	► Children aged 0-6 years not attending an early childcare facility
		► Patient samples (children with specific conditions/diseases)
		► Older age groups (e.g., school children, adolescents, adults, elderly people)
Factors of interest	Compositional characteristics at the early childcare-level:	Compositional characteristics outside the early childcare- level:
	► Gender	► At the family level
	► Age	► In the home environment
	► Immigrant background	► In other institutions (e.g., in schools)
	► Language skills	
	► Socioeconomic position	
	► Parental commitment	
	Contextual characteristics at the early childcare level:	Contextual characteristics outside the early childcare-level:
	► Location of childcare facility	► At the family level
	► Type of childcare facility	► In the home environment
	► Childcare facility size	► In other institutions (e.g., in schools)
	► Group size	
	► Duration of childcare (full-time, half-time)	
	► Teacher/child ratio	
	► Staff characteristics (e.g., number, age, sex, migration background, qualification)	
	► Toys/playing equipment	
	► Financial resources	
	► Opportunities for PA (e.g., sport rooms, outdoor area, playground)	
	► Equipment for PA	
	► Integration of PA in daily routines	
	► Projects that promote PA	
	► Resources for healthy eating	
	► Cooking facilities	
	► Lunch/other meals offered	
	► Food quality	
	► Free access to water/food	
	► Nutrition rules (e.g., lunch box content)	
	► Projects that promote healthy eating	
Outcomes	► Health outcomes (e.g., self-rated health, physical health, mental health)	
	► Health behavior (e.g., nutrition, PA sedentary behavior, media consumption, passive smoke exposure)	
	► Other health-related outcomes (e.g., obesity, wellbeing, quality of life)	
Regions/countries	► Developed countries	► Developing countries
		► Countries in transition
Languages	► English	► All other languages
	► German	

**Table A2:** Reported associations or moderations between family SEP with outcomes at the ECEC center level.

Key Findings
Household income was positively and significantly related to child’s BMI [94].
The higher the mother’s education, the less is the screen time of the child during child care [117].
Education of mother is correlated with impulsivity, re-reading skills, and pre-math skills [104].
Parental education level was significantly different across naptime groups: education level was higher in the 0–60 min group than in the <60min groups (maybe an effect of the different program types) [111].
Children of higher SEP families showed more positively adaptive behaviors compared with low- SEP peers.High SEP was negatively related to depression, inattention, externalizing, and positively to peer relationships, academic competence, and prosocial behavior.Family SEP moderated the association of social position with adaptive child outcomes. Specifically, family SES significantly moderated the relation between rank and prosociality, with subordinate, low-SES children having the lowest levels of prosocial behavior [29].
